# ﻿Molecular phylogeny and taxonomy reveal two new genera and five new species in Phanerochaetaceae (Polyporales) from Yunnan, Southwest China

**DOI:** 10.3897/mycokeys.113.140624

**Published:** 2025-02-12

**Authors:** Ying Xu, Yang Yang, Xin Yang, Daxiang Chen, Wen Zheng, Kaize Shen, Sicheng Zhang, Changlin Zhao

**Affiliations:** 1 College of Forestry, Southwest Forestry University, Kunming 650224, China; 2 Tongbiguan Provincial Nature Reserve, Mangshi 678499, China; 3 Key Laboratory of Forest Disaster Warning and Control in Universities of Yunnan Province, Southwest Forestry University, Kunming 650224, China

**Keywords:** Biodiversity, fungal classification, new taxa, wood-inhabiting fungi, Yunnan Province

## Abstract

In the present study, two new genera *Paradonkia*, and *Neodonkiella*, and five new species, *viz. Paradonkiafarinacea*, *Neodonkiellayinjiangensis*, *Phanerochaetealbocremea*, *Phanerochaetefissurata*, and *Phanerochaetepunctata* collected from southern China, are proposed based on a combination of morphological features and molecular evidence. *Paradonkiafarinacea* is characterized by the resupinate, membranaceous basidiomata with pale cream to gray cream hymenial surface and a monomitic hyphal system with simple septa and clamp connections; *Neodonkiellayinjiangensis* is characterized by soft coriaceous basidiomata, a monomitic hyphal system and ellipsoid basidiospores (3.5–5 × 2–2.5 µm); *Phanerochaetealbocremea* is characterized by resupinate basidiomata with white to a pale cream hymenial surface, and ellipsoid basidiospores (3.5–5 × 2–3 µm); *Phanerochaetefissurata* is characterized by gray-brown and cracked hymenial surface, and ellipsoid basidiospores (4–5.5 × 2–3 µm) and *Phanerochaetepunctata* is characterized by farinaceous basidiomata, a monomitic hyphal system, and ellipsoid basidiospores. Sequences of the internal transcribed spacers (ITS) and the large subunit (nLSU) of the nuclear ribosomal DNA (rDNA) markers of the studied samples were generated. Phylogenetic analyses were performed using the maximum likelihood, maximum parsimony, and Bayesian inference methods. The phylogram based on the ITS+nLSUrDNA gene regions, revealed that two new genera, *Paradonkia* and *Neodonkiella*, belong to the family Phanerochaetaceae, and three new species belong to the genus *Phanerochaete* in the family Phanerochaetaceae.

## ﻿Introduction

fungi, as eukaryotic microorganisms, are pivotal in ecological ecosystems, serving as decomposers and mutualists of both dead and living plants and animals. They are key players in carbon cycling in forest soils, mediating the mineral nutrition of plants, and alleviating the carbon limitations of other soil organisms (Cui et al. 2019; James et al. 2020; Liu et al. 2023; Zhao et al. 2023b). Wood-inhabiting fungi, with their distinct and diverse characteristics, form an ecologically important branch of the tree of life, further underlining their significance (Dai et al. 2021; Yang et al. 2024).

The family Phanerochaetaceae Jülich, belonging to the order Polyporales (Basidiomycota), was typified by *Phanerochaete* P. Karst. Twenty-five genera were placed in this family Phanerochaetaceae as *Alboefibula* C.C. Chen & Sheng H. Wu, *Bjerkandera* P. Karst., *Callosus* C.L. Zhao, *Cremeoderma* Sheng H. Wu & C.C. Chen, *Crepatura* C.L. Zhao, *Donkia* Pilát, *Donkiella* J.H. Dong & C.L. Zhao, *Efibulella* Zmitr., *Gelatinofungus* Sheng H. Wu, et al., *Geliporus* Yuan Yuan, et al., *Hapalopilus* P. Karst., *Hyphodermella* J. Erikss. & Ryvarden, *Odontoefibula* C.C. Chen & Sheng H. Wu, *Oxychaete* Miettinen, *Phaeophlebiopsis* Floudas & Hibbett, *Phanerina* Miettinen, *Phanerochaete* P. Karst., *Phlebiopsis* Jülich, *Pirex* Hjortstam & Ryvarden, *Porostereum* Pilát, *Quasiphlebia* C.C. Chen & Sheng H. Wu, *Rhizochaete* Gresl., Nakasone & Rajchenb., *Riopa* D.A. Reid, *Roseograndinia* Hjortstam & Ryvarden and *Terana* Adans according to recent studies (Dong et al. 2024; He et al. 2024). In Phanerochaetaceae morphology, the corticioid species are predominant, along with a few resupinate polypores and hydnaceous species (Chen et al. 2021). The hyphal system of this family is usually monomitic, rarely dimitic, and the generative hyphae are usually simple septa, rarely nodose-septate, and cystidia are often present, and basidiospores are usually thin-walled, smooth, and colorless (Justo et al. 2017; Chen et al. 2021).

The genus *Phanerochaete* P. Karst., belonging to the family Phanerochaetaceae (Polyporales, Basidiomycota), was typified by *P.alnea* (Fr.) P. Karst (Deng et al. 2024). It is characterized by the membranaceous, smooth hymenial surface (some are tuberculate, odontioid-hydnoid, or merulioid-poroid), mostly monomitic hyphal system, simple septa generative hyphae or with rare clamp connections in the subiculum, clavate basidia and ellipsoid to cylindrical, thin-walled and smooth basidiospores, which are inamyloid and non-dextrinoid (Wu et al. 2018). The colorless subiculum is present in most species, but a brownish subiculum also occurs (Chen et al. 2021). Based on the MycoBank database (http://www.MycoBank.org, accessed on 06 January 2025) and the Index Fungorum (www.indexfungorum.org; accessed on 06 January 2025), 208 names are registered in the genus *Phanerochaete* but 121 species have been accepted worldwide (Chen et al. 2021; Wang and Zhao 2021; Yu et al. 2023; Deng et al. 2024; Dong et al. 2024; Luo et al. 2024).

During investigations on wood-inhabiting fungi in the Yunnan-Guizhou Plateau, China, many wood-inhabiting fungal specimens were collected. To clarify the placement and relationships of these specimens, we carried out a phylogenetic and taxonomic study based on the ITS+nLSU sequences. These specimens were assigned to the family Phanerochaetaceae. Therefore, two new genera, *Paradonkia*, and *Neodonkiella*, and five new species, *Paradonkiafarinacea*, *Neodonkiellayinjiangensis*, *Phanerochaetealbocremea*, *Phanerochaetefissurata*, and *Phanerochaetepunctata* are proposed with descriptions, and illustrations, and phylogenetic analysis results.

## ﻿Materials and methods

### ﻿Sample collection and herbarium specimen preparation

The fresh fruiting bodies were collected on the fallen angiosperm branches and stumps and dead bamboo from Yunnan Province, China. The samples were photographed in situ, and important collection information was noted (Rathnayaka et al. 2024) and macroscopic characteristics were recorded. Photographs were recorded by a Nikon D7100 camera. All the photos were focus-stacked using Helicon Focus software. Macroscopic details were recorded and transported to a field station where the fruit body was dried on an electronic food dryer at 40 °C (Hu et al. 2022), and once dried, the specimens were sealed in an envelope and zip-lock plastic bags and labelled (Zhao et al. 2023a). The dried specimens were deposited in the Herbarium of the Southwest Forestry University (SWFC), Kunming, Yunnan Province, China.

### ﻿Morphology

The macromorphological descriptions were based on field notes and photos captured in the field and lab. The color terminology follows Petersen (1996). The micromorphological data were obtained from the dried specimens after observation under a light microscope with a magnification of 10 × 100 oil (Zhao et al. 2023a). Sections were mounted in 5% potassium hydroxide (KOH) and Congo red solution, and we also used other reagents, including Cotton Blue and Melzer’s reagent, to observe micromorphology following previous studies (Moreno et al. 2017; Dong et al. 2024; Wang et al. 2024). To show the variation in spore sizes, 5% of measurements were excluded from each end of the range and shown in parentheses. At least thirty basidiospores from each specimen were measured. Stalks were excluded from basidia measurements and the hilar appendage was excluded from basidiospores measurements. The following abbreviations are used: KOH = 5% potassium hydroxide water solution, CB = Cotton Blue, CB– = acyanophilous, IKI = Melzer’s reagent, IKI– = both inamyloid and indextrinoid, L = mean spore length (arithmetic average for all spores), W = mean spore width (arithmetic average for all spores), Q = variation in the L/W ratios between the specimens studied, and n = a/b (number of spores (a) measured from a given number (b) of specimens).

### ﻿Molecular phylogeny

The CTAB rapid plant genome extraction kit-DN14 (Aidlab Biotechnologies Co., Ltd., Beijing, China) was used to obtain genomic DNA from the dried specimens according to the manufacturer’s instructions. The ITS region was amplified with ITS5 and ITS4 primers (White et al. 1990). The nLSU region was amplified with the LR0R and LR7 (Vilgalys and Hester 1990). The PCR procedure for ITS was as follows: initial denaturation at 95 °C for 3 min, followed by 35 cycles at 94 °C for 40 s, 58 °C for 45 s and 72 °C for 1 min, and a final extension of 72 °C for 10 min. The PCR procedure for nLSU was as follows: initial denaturation at 94 °C for 1 min, followed by 35 cycles at 94 °C for 30 s, 48 °C for 1 min, and 72 °C for 1.5 min, and a final extension of 72 °C for 10 min. The PCR products were purified and sequenced at Kunming Tsingke Biological Technology Limited Company (Yunnan Province, P.R. China). The newly generated sequences were deposited in NCBI GenBank (Table 1).

**Table 1.** Names, voucher numbers, localities, references, and corresponding GenBank accession numbers of the taxa used in this study. [New species are shown in bold; * refers to type material].

Species Name	Sample No.	GenBank Accession No.			References
		ITS	nLSU	Country	
*Alboefibulabambusicola*	Chen 2304	MZ636926	MZ637091	China	Chen et al. (2021)
*Alboefibulagracilis*	Wu 1809-106	MZ636929	MZ637094	China	Chen et al. (2021)
*Artomycesniveus*	CLZhao 19094	OR094479	OR461459	China	Dong et al. (2024)
*Bjerkanderaadusta*	HHB-12826-Sp	KP134983	KP135198	USA	Floudas and Hibbett (2015)
*Bjerkanderacentroamericana*	L-13104-sp	KY948791	KY948855	Costa Rica	Justo et al. (2017)
*Callosuswenshanensis*	CLZhao 16017	MW553934	MW553936	China	Chen et al. (2022)
*Callosuswenshanensis*	CLZhao 16034	MW553935	MW553937	China	Chen et al. (2022)
*Cremeodermaunicum*	Wu 1707-94	MZ636939	MZ637102	China	Chen et al. (2021)
*Cremeodermaunicum*	Wu 1707-100	MZ636938	MZ637101	China	Chen et al. (2021)
*Crepaturaellipsospora*	CLZhao 1265	MK343692	MK343696	China	Ma and Zhao (2019)
*Crepaturaellipsospora*	CLZhao 1260	MK343693	MK343697	China	Ma and Zhao (2019)
*Crepaturaellipsospora*	CLZhao 126	MK343692	MK343696	China	Ma and Zhao (2019)
*Donkiapulcherrima*	GC 1707-11	LC378994	LC379152	China	Chen et al. (2018)
*Donkiapulcherrima*	Gothenburg-2022	KX752591	KX752591	Austria	Miettinen et al. (2016)
*Donkiellayunnanensis*	CLZhao 3931	OR094482	OR461467	China	Dong et al. (2024)
*Donkiellayunnanensis*	CLZhao 18292	OR094483	OR461468	China	Dong et al. (2024)
*Efibulelladeflectens*	FCUG 1568	AF141619	AF141619	Sweden	Parmasto and Hallenberg (2000)
*Gelatinofungusbrunneus*	GC 1703-31	LC387339	LC387344	China	Chen et al. (2018)
*Gelatinofungusbrunneus*	Wu 1207-162	MZ636978	MZ637139	China	Chen et al. (2021)
*Geliporusexilisporus*	Dai 2172	KU598211	KU598216	China	Yuan et al. (2017)
*Geliporusexilisporus*	GC 1702-15	LC378995	LC379153	China	Chen et al. (2018)
*Hapalopiluspercoctus*	H 7008581	KX752597	KX752597	Botswana	Miettinen et al. (2016)
*Hapalopilusrutilans*	FP-102473-Sp	MZ636981	MZ637142	USA	Chen et al. (2021)
*Hyphodermellacorrugata*	MA-fungi 24238	FN600378	JN939586	Portugal	Telleria et al. (2010)
*Hyphodermellarosae*	GC 1604-113	MZ636986	MZ637147	China	Chen et al. (2021)
*Odontoefibulaorientalis*	Wu 0910-57	LC363490	LC363495	China	Chen et al. (2018)
*Odontoefibulaorientalis*	GC 1703-76	LC379004	LC379156	China	Chen et al. (2018)
*Oxychaetecervinogilva*	GC 1501-16	MZ422783	MZ637173	China	Chen et al. (2021)
*Oxychaetecervinogilva*	Dmitry Schigel 5216	KX752596	KX752596	Australia	Chen et al. (2021)
***Paradonkiafarinacea***	CLZhao 27184*	**PQ527890**	**PQ527887**	**China**	**Present study**
***Paradonkiafarinacea***	**CLZhao 27221**	**PQ527891**	**PQ527888**	**China**	**Present study**
***Neodonkiellayinjiangensis***	CLZhao 30585*	**PQ527892**	**PQ527889**	**China**	**Present study**
*Phaeophlebiopsiscaribbeana*	HHB-6990	KP135415	KP135243	USA	Floudas and Hibbett (2015)
*Phaeophlebiopsispeniophoroides*	FP-150577	KP135417	KP135273	USA	Floudas and Hibbett (2015)
*Phanerinamellea*	Wu 1010-34	MZ422784	MZ637176	China	Chen et al. (2021)
*Phanerinamellea*	WEI 17-224	LC387333	LC387340	China	Chen et al. (2018)
*Phanerochaeteaculeata*	Wu 1809-278	MZ422786	MZ637178	China	Chen et al. (2021)
*Phanerochaeteaculeata*	GC 1703-117	MZ422785	MZ637177	China	Chen et al. (2021)
*Phanerochaetealbida*	WEI 18-365	MZ422789	MZ637180	China	Chen et al. (2021)
*Phanerochaetealbida*	GC 1407-14	MZ422788	MZ637179	China	Chen et al. (2021)
***Phanerochaetealbocremea***	**CLZhao 31998**	**PQ454009**	**PQ454675**	**China**	**Present study**
***Phanerochaetealbocremea***	**CLZhao 32032**	**PQ454010**	**PQ454676**	**China**	**Present study**
***Phanerochaetealbocremea***	**CLZhao 32035**	**PQ454011**	**PQ454677**	**China**	**Present study**
***Phanerochaetealbocremea***	CLZhao 32235*	**PQ454012**	—	**China**	**Present study**
*Phanerochaetealnea*	Larsson 12054	KX538924	—	Norway	Spirin et al. (2017)
*Phanerochaetealpina*	Wu 1308-61	MZ422790	MZ637182	China	Chen et al. (2021)
*Phanerochaetealpina*	Wu 1308-77	MZ422791	MZ637183	China	Chen et al. (2021)
*Phanerochaetearizonica*	RLG-10248-Sp	KP135170	KP135239	USA	Floudas and Hibbett (2015)
*Phanerochaeteaustralis*	He 6013	MT235656	MT248136	China	Phookamsak et al. (2019)
*Phanerochaeteaustralis*	HHB-7105-Sp	KP135081	KP135240	USA	Floudas and Hibbett (2015)
*Phanerochaeteaustralosanguinea*	MA:fungi:91308	MH233925	MH233928	Chile	Phookamsak et al. (2019)
*Phanerochaeteaustralosanguinea*	MA:fungi:91309	MH233926	MH233929	Chile	Phookamsak et al. (2019)
*Phanerochaetebambusicola*	He 3606	MT235657	MT248137	China	Xu et al. (2020)
*Phanerochaetebambusicola*	Wu 0707-2	MF399404	MF399395	China	Wu et al. (2017)
*Phanerochaetebrunnea*	He 4192	MT235658	MT248138	China	Xu et al. (2020)
*Phanerochaeteburdsallii*	He 2066	MT235690	MT248177	USA	Xu et al. (2020)
*Phanerochaeteburtii*	HHB-4618-Sp	KP135117	KP135241	USA	Floudas and Hibbett (2015)
*Phanerochaeteburtii*	FD-171	KP135116	—	USA	Floudas and Hibbett (2015)
*Phanerochaetecalotricha*	Vanhanen382	KP135107	—	Finland	Floudas and Hibbett (2015)
*Phanerochaetecanobrunnea*	He 5726	MT235659	MT248139	SriLanka	Wu et al. (2017)
*Phanerochaetecanobrunnea*	CHWC1506-66	LC412095	LC412104	China	Xu et al. (2020)
*Phanerochaetecarnosa*	He 5172	MT235660	MT248140	China	Xu et al. (2020)
*Phanerochaetecarnosa*	HHB-9195	KP135129	KP135242	USA	Floudas and Hibbett (2015)
*Phanerochaetechrysosporium*	He 5778	MT235661	MT248141	SriLanka	Xu et al. (2020)
*Phanerochaetechrysosporium*	HHB-6251-Sp	KP135094	KP135246	USA	Floudas and Hibbett (2015)
*Phanerochaetecinerea*	He 5998	—	MT248171	China:	Xu et al. (2020)
*Phanerochaetecinerea*	He 6003	—	MT248172	China	Xu et al. (2020)
*Phanerochaetecitrinosanguinea*	FP-105385-Sp	KP135100	—	USA	Floudas and Hibbett (2015)
*Phanerochaetecitrinosanguinea*	FD-287	KP135095	—	USA	Floudas and Hibbett (2015)
*Phanerochaeteconcrescens*	He 4657	MT235662	MT248142	China	Chen et al. (2021)
*Phanerochaeteconcrescens*	Spirin 7322	KP994380	KP994382	Russia	Volobuev et al. (2015)
*Phanerochaetecrystallina*	Chen 3823	MZ422802	—	China	Chen et al. (2021)
*Phanerochaetecrystallina*	Chen 3576	MZ422801	—	China	Chen et al. (2021)
*Phanerochaetecumulodentata*	He 2995	MT235664	MT248144	China	Phookamsak et al. (2019)
*Phanerochaetecumulodentata*	LE<RUS>:298935	KP994359	KP994386	Russia	Volobuev et al. (2015)
*Phanerochaetecystidiata*	He 4224	MT235665	MT248145	China	Xu et al. (2020)
*Phanerochaetecystidiata*	Wu 1708-326	LC412097	LC412100	China	Wu et al. (2018)
*Phanerochaeteericina*	HHB-2288	KP135167	KP135247	USA	Floudas and Hibbett (2015)
*Phanerochaeteericina*	He 4285	MT235666	MT248146	China	Xu et al. (2020)
***Phanerochaetefissurata***	CLZhao 35311*	**PQ454013**	**PQ454678**	**China**	**Present study**
***Phanerochaetefissurata***	**CLZhao 35321**	**PQ454014**	**PQ454679**	**China**	**Present study**
*Phanerochaetefusca*	Wu1409-163	LC412099	LC412106	China	Wu et al. (2018)
*Phanerochaetefusca*	Wu 1409-161	LC412098	LC412105	China:	Wu et al. (2018)
*Phanerochaetegranulata*	Chen 2835	MZ422808	MZ637194	China	Chen et al. (2021)
*Phanerochaetegranulata*	GC 1703-5	MZ422809	MZ637195	China	Chen et al. (2021)
*Phanerochaetegranulata*	Wu 9210-57	MZ422810	MZ637196	China	Chen et al. (2021)
*Phanerochaeteguangdongensis*	Wu 1809-348	MZ422813	MZ637199	China	Chen et al. (2021)
*Phanerochaeteguangdongensis*	Wu 1809-319	MZ422811	MZ637197	China	Chen et al. (2021)
*Phanerochaetehainanensis*	He 3562	MT235692	MT248179	China	Boonmee et al. (2021)
*Phanerochaetehymenochaetoides*	He 5988	—	MT248173	China	Xu et al. (2020)
*Phanerochaeteincarnata*	He 20120728-1	MT235669	MT248149	China	Xu et al. (2020)
*Phanerochaeteincarnata*	WEI 16-075	MF399406	MF399397	China	Wu et al. (2017)
*Phanerochaetelaevis*	He 20120917-8	MT235670	MT248150	China	Xu et al. (2020)
*Phanerochaetelaevis*	HHB-15519	KP135149	KP135249	USA	Floudas and Hibbett (2015)
*Phanerochaeteleptocystidiata*	He 5853	MT235685	MT248168	China	Xu et al. (2020)
*Phanerochaeteleptocystidiata*	Dai 10468	MT235684	MT248167	China	Xu et al. (2020)
*Phanerochaetelivescens*	He 5010	MT235671	MT248151	China	Xu et al. (2020)
*Phanerochaetemetuloidea*	He 2766	MT235682	MT248164	China	Xu et al. (2020)
*Phanerochaeteminor*	He 3988	MT235686	MT248170	China	Xu et al. (2020)
*Phanerochaetemopanshanensis*	CLZhao 2357	OR096190	OR461450	China	Dong et al. (2024)
*Phanerochaeteparmastoi*	He 4570	MT235673	MT248153	China	Xu et al. (2020)
*Phanerochaeteparmastoi*	Wu 880313-6	MZ422823	GQ470654	China	Chen et al. (2021)
*Phanerochaeteporostereoides*	He 1902	KX212217	KX212221	China	Liu and He (2016)
*Phanerochaeteporostereoides*	He 1908	KX212218	KX212222	China	Liu and He (2016)
*Phanerochaetepruinosa*	CLZhao 7112	MZ435346	MZ435350	China	Wang and Zhao (2021)
*Phanerochaetepruinosa*	CLZhao 7113	MZ435347	MZ435351	China	Wang and Zhao (2021)
*Phanerochaetepseudosanguinea*	FD-244	KP135098	KP135251	USA	Floudas and Hibbett (2015)
***Phanerochaetepunctata***	**CLZhao 30365**	**PQ454015**	**PQ454680**	**China**	**Present study**
***Phanerochaetepunctata***	CLZhao 30512*	**PQ454016**	**PQ454681**	**China**	**Present study**
*Phanerochaeterhizomorpha*	GC 1708-335	MZ422824	MZ637208	China	Chen et al. (2021)
*Phanerochaeterhizomorpha*	GC 1708-354	MZ422825	MZ637209	China	Chen et al. (2021)
*Phanerochaeterhodella*	FD-18	KP135187	KP135258	USA	Floudas and Hibbett (2015)
*Phanerochaeterobusta*	Wu 1109-69	MF399409	MF399400	China	Wu et al. (2018)
*Phanerochaeterobusta*	MG265	KP127068	KP127069	China	Ghobad-Nejhad et al. (2015)
*Phanerochaetesanguineocarnosa*	FD-359	KP135122	KP135245	USA	Floudas and Hibbett (2015)
*Phanerochaetesanguineocarnosa*	FD-528	KP135121	—	USA	Floudas and Hibbett (2015)
*Phanerochaetesinensis*	He 4660	MT235688	MT248175	China	Xu et al. (2020)
*Phanerochaetesinensis*	GC1809-56	MT235689	MT248176	China	Xu et al. (2020)
*Phanerochaetesordida*	FD-241	KP135136	KP135252	USA	Floudas and Hibbett (2015)
*Phanerochaetespadicea*	Wu 0504-15	MZ422837	MZ637219	China	Chen et al. (2021)
*Phanerochaetespadicea*	Wu 0504-11	MZ422836	—	China	Chen et al. (2021)
*Phanerochaetestereoides*	He 5824	MT235677	MT248158	SriLanka	Xu et al. (2020)
*Phanerochaetestereoides*	He 2309	KX212219	KX212223	China	Liu and He (2016)
*Phanerochaetesubcarnosa*	Wu 9310-3	MZ422841	GQ470642	China	Chen et al. (2021)
*Phanerochaetesubcarnosa*	GC 1809-90	MZ422840	MZ637222	China	Chen et al. (2021)
*Phanerochaetesubceracea*	FP-105974-R	KP135162	KP135255	USA	Floudas and Hibbett (2015)
*Phanerochaetesubceracea*	HHB-9434	KP135163	—	USA	Floudas and Hibbett (2015)
*Phanerochaetesubrosea*	He 2421	MT235687	MT248174	China	Xu et al. (2020)
*Phanerochaetesubsanguinea*	CLZhao 10470	MZ435348	MZ435352	China	Wang and Zhao (2021)
*Phanerochaetesubsanguinea*	CLZhao 10477	MZ435349	MZ435353	China	Wang and Zhao (2021)
*Phanerochaetesubtropica*	CLZhao F8716	OP605486	OQ195089	China	Yu et al. (2023)
*Phanerochaetesubtropica*	CLZhao F2763	OP605518	OQ195090	China	Yu et al. (2023)
*Phanerochaetesubtuberculata*	CLZhaoF5130	OP605484	OQ195088	China	Yu et al. (2023)
*Phanerochaetesubtuberculata*	CLZhaoF6838	OP605485	OQ195087	China	Yu et al. (2023)
*Phanerochaetetaiwaniana*	He 5269	MT235680	MT248161	Vietnam	Xu et al. (2020)
*Phanerochaetetaiwaniana*	Wu 0112-13	MF399412	MF399403	China	Chen et al. (2021)
*Phanerochaetetongbiguanensis*	CLZhao 30606	OR917875	OR921222	China	Deng et al. (2024)
*Phanerochaetevelutina*	He 3079	MT235681	MT248162	China	Xu et al. (2020)
*Phanerochaetevelutina*	Kotiranta 25567	KP994354	KP994387	Russia	Volobuev et al. (2015)
*Phanerochaeteyunnanensis*	He 2719	MT235683	MT248166	China	Xu et al. (2020)
*Phanerochaeteyunnanensis*	He 2697	—	MT248165	China	Xu et al. (2020)
*Phlebiopsisgigantea*	FP-70857	KP135390	KP135272	USA	Floudas and Hibbett (2015)
*Phlebiopsiscrassa*	KKN-86	KP135394	KP135215	USA	Floudas and Hibbett (2015)
*Phlebiopsisgalochroa*	FP-102937	KP135391	KP135270	USA	Justo et al. 2017
*Pirexconcentricus*	Kropp160Bup6-R	KP134985	—	USA	Floudas and Hibbett (2015)
*Pirexconcentricus*	OSC-41587	KP134984	KP135275	USA	Floudas and Hibbett (2015)
*Porostereumfulvum*	LY:18491	MG649452	MG649454	France	Unpublished
*Porostereumspadiceum*	Wu 9508-139	MZ637062	MZ637263	China	Chen et al. (2021)
*Quasiphlebiadensa*	WEI 17-057	MZ637066	MZ637265	USA	Chen et al. (2021)
*Quasiphlebiadensa*	Wu 9304-33	MZ637067	MZ637266	China	Chen et al. (2021)
*Rhizochaetefilamentosa*	HHB-3169	KP135410	KP135278	USA	Floudas and Hibbett (2015)
*Rhizochaeteradicata*	FD-123	KP135407	KP135279	USA	Floudas and Hibbett (2015)
*Riopametamorphosa*	Spirin 2395	KX752601	KX75260	Russia	Miettinen et al. (2016)
*Riopapudens*	Dai 19241	OL470307	OL462822	China	Unpublished
*Roseograndiniajilinensis*	Wu 1307-137	MZ637077	MZ637275	China	Chen et al. (2021)
*Roseograndiniaminispora*	WEI 18-511	MZ637079	MZ637277	China	Chen et al. (2021)
*Teranacaerulea*	FP-104073	KP134980	KP135276	USA	Floudas and Hibbett (2015)
*Teranacaerulea*	GC 1507-2	MZ637090	MZ637287	China	Chen et al. (2021)

**Figure 1.**
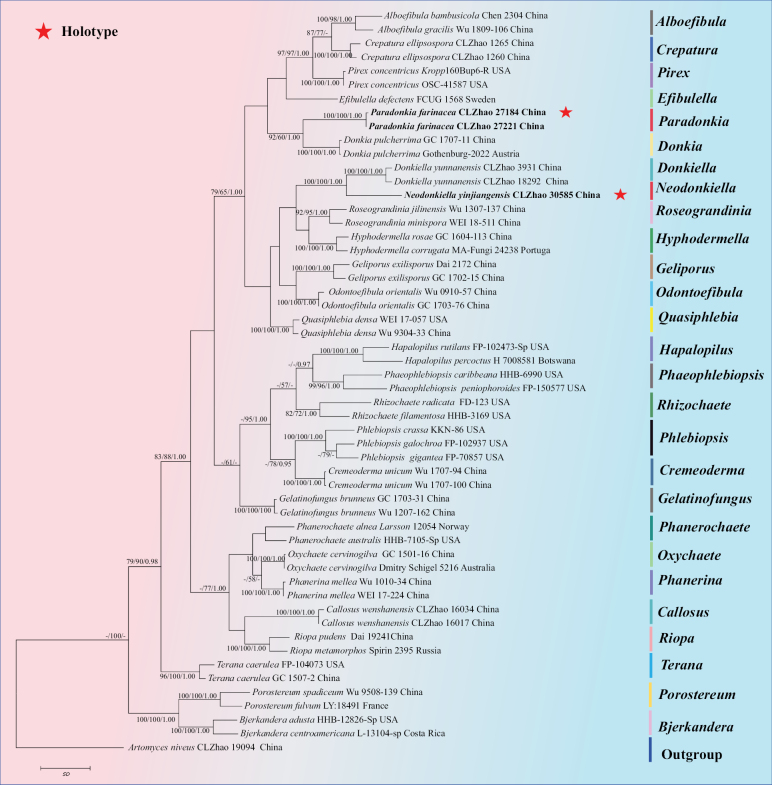
Maximum parsimony strict consensus tree illustrating the phylogeny of *Paradonkia* and *Neodonkiella* and related genera in the family Phanerochaetaceae based on ITS+nLSU sequences. Branches are labelled with maximum likelihood bootstrap value ≥ 70%, parsimony bootstrap value ≥ 50%, and Bayesian posterior probabilities ≥ 0.95. Colored bars represent different genera.

**Figure 2.**
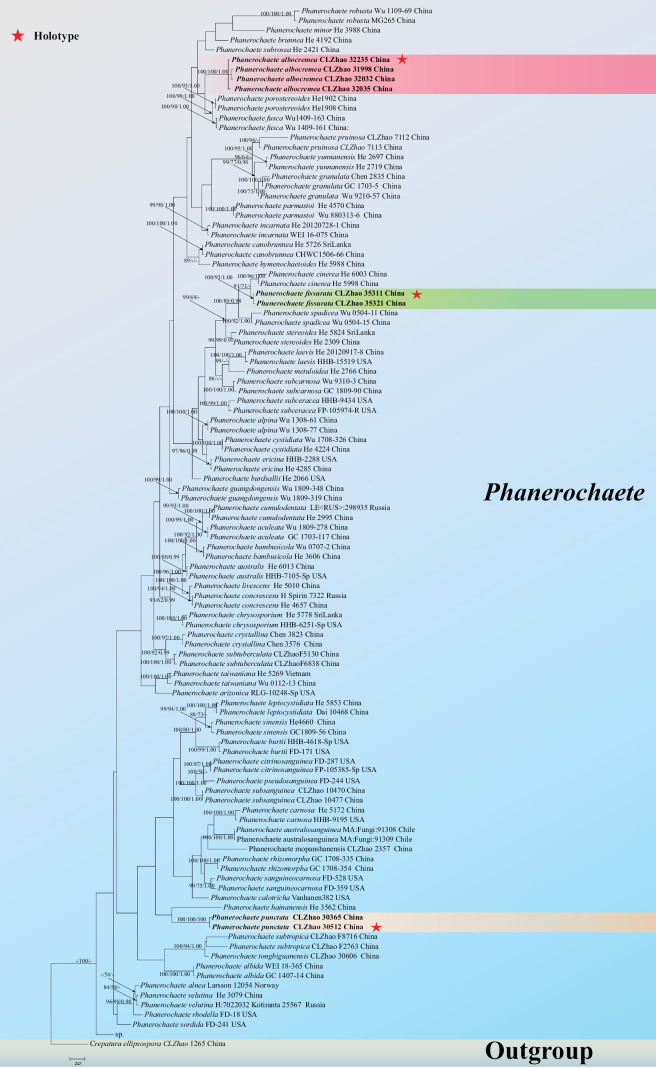
Maximum parsimony strict consensus tree illustrating the phylogeny of three new species and related genera in the genus *Phanerochaete* based on ITS+nLSU sequences. Branches are labelled with maximum likelihood bootstrap value ≥ 70%, parsimony bootstrap value ≥ 50%, and Bayesian posterior probabilities ≥ 0.95.

The sequences were aligned in MAFFT v. 7 using the G-INS-i strategy (Katoh et al. 2019). The alignment was adjusted manually using AliView v. 1.27 (Larsson 2014). The dataset was aligned first, and then the sequences of ITS+nLSU were combined with Mesquite v. 3.51. The combined ITS+nLSU sequences were used to infer the position of the new species and closely related species. The sequence of *Artomycesniveus* J.H. Dong & C.L. Zhao obtained from GenBank was used as an outgroup to root trees in the ITS+nLSU analysis (Fig. 1) in the family Phanerochaetaceae (Dong et al. 2024). The sequence of *Crepaturaellipsospora* C.L. Zhao obtained from GenBank was used as an outgroup to root trees in the ITS+nLSU analysis (Fig. 2) in the genus *Phanerochaete* (Ma and Zhao 2019).

Maximum Parsimony (MP), Maximum Likelihood (ML), and Bayesian Inference (BI) analyses were applied to the combined three datasets following a previous study (Zhao and Wu 2017) and the tree construction procedure was performed in PAUP* v. 4.0b10 (Swofford 2002). All characters were equally weighted, and gaps were treated as missing data. Trees were inferred using the heuristic search option with TBR branch swapping and 1000 random sequence additions. Max trees were set to 5000, branches of zero length were collapsed, and all parsimonious trees were saved. Clade robustness was assessed using bootstrap (BT) analysis with 1000 replicates (Felsenstein 1985). Descriptive tree statistics, tree length (TL), consistency index (CI), retention index (RI), rescaled consistency index (RC), and homoplasy index (HI) were calculated for each maximum parsimonious tree generated. The multiple sequence alignment was also analyzed using Maximum Likelihood (ML) in RAxML-HPC2 on XSEDE v. 8.2.8 with default parameters (Miller et al. 2012). Branch support (BS) for ML analysis was determined by 1000 bootstrap replicates.

jModelTest v. 2 (Darriba et al. 2012) was used to determine the best-fit evolution model for each dataset for the purposes of Bayesian Inference (BI), which was performed using MrBayes 3.2.7a with a GTR+I+G model of DNA substitution and a gamma distribution rate variation across sites (Ronquist et al. 2012). The first one-quarter of all the generations were discarded as burn-in. The majority-rule consensus tree of all the remaining trees was calculated. Branches were considered significantly supported if they received a Maximum Likelihood bootstrap value (BS) of ≥ 70%, a Maximum Parsimony bootstrap value (BT) of ≥ 50%, or Bayesian Posterior Probabilities (BPP) of ≥ 0.95.

## ﻿Results

### ﻿Molecular phylogeny

The Phanerochaetaceae aligned dataset comprised 54 specimens representing 28 species. Four Markov chains were run for two runs from random starting trees, each for three million generations for the combined ITS+nLSU (Fig. 1) dataset with trees and parameters sampled every 1,000 generations. The dataset had an aligned length of 2,205 characters, of which 1,536 characters are constant, 191 are variable and parsimony uninformative, and 478 are parsimony informative. Maximum Parsimony analysis yielded one equally parsimonious tree (TL = 2,599, CI = 0.4055, HI = 0.5945, RI = 0.5984 and RC = 0.2427). The best model for the ITS+nLSU dataset, estimated and applied in the Bayesian analysis, was GTR+I+G. Both Bayesian analysis and ML analysis resulted in a similar topology to MP analysis with an average standard deviation of split frequencies = 0.006800 (BI), and the effective sample size (ESS) for Bayesian analysis across the two runs is double the average ESS (avg. ESS) = 294.

The *Phanerochaete* aligned dataset comprised 107 specimens representing 59 species. Four Markov chains were run for two runs from random starting trees, each for 8.5 million generations for the ITS+nLSU (Fig. 2) dataset, with trees and parameters sampled every 1,000 generations. The dataset had an aligned length of 2,333 characters, of which 1,578 characters are constant, 255 are variable, parsimony uninformative, and 500 are informative. Maximum Parsimony analysis yielded one equally parsimonious tree (TL = 2,872, CI = 0.3729, HI = 0.6271, RI = 0.5891 and RC = 0.2197). The best model for the ITS dataset, estimated and applied in the Bayesian analysis, was GTR+I+G. Both Bayesian analysis and ML analysis resulted in a similar topology to MP analysis with an average standard deviation of split frequencies = 0.012119 (BI), and the effective sample size (ESS) for Bayesian analysis across the two runs is double the average ESS (avg. ESS) = 256.

The phylogram, based on the combined ITS+nLSU sequences (Fig. 1) analysis, showed that two new genera, *Paradonkia* and *Neodonkiella* were assigned to the family Phanerochaetaceae. The phylogenetic tree, based on ITS+nLSU sequences (Fig. 2), revealed that *Phanerochaetealbocremea* formed a monophyletic lineage and was closely related to *Phanerochaeteporostereoides* S.L. Liu & S.H. He and *Phanerochaetefusca* Sheng H. Wu et al. The new species *Phanerochaetefissurata* was retrieved as a sister to *Phanerochaetecinerea* Y.L. Xu & S.H. He. The new taxon *Phanerochaetepunctata* was sister to *Phanerochaetehainanensis* S.H. He & Y.C. Dai.

### ﻿Taxonomy

#### ﻿ Phanerochaetaceae

Taxon classificationFungiPolyporalesPhanerochaetaceae

Jülich

E66AB9CA-3C5A-5148-AF3C-387289D7213C

##### Type genus.

*Phanerochaete* P. Karst.

##### Description.

Mostly corticioid species, along with a few resupinate or pileate polypores (Wu et al. 2022a; Zhao et al. 2024), and hydnaceous species; hyphal system usually monomitic, rarely dimitic; hyphae usually simple septate, rarely nodose septate; basidiospores thin-walled, smooth, colorless; cystidia often present. Producing a white rot (Chen et al. 2021).

Accepted genera. *Alboefibula*, *Bjerkandera*, *Callosus*, *Cremeoderma*, *Crepatura*, *Donkia*, *Donkiella*, *Efibulella*, *Gelatinofungus*, *Geliporus*, *Hapalopilus*, *Hyphodermella*, *Odontoefibula*, *Oxychaete*, *Paradonkia*, *Neodonkiella*, *Phanerina*, *Phanerochaete*, *Phaeophlebiopsis*, *Phlebiopsis*, *Pirex*, *Porostereum*, *Quasiphlebia*, *Rhizochaete*, *Riopa*, *Roseograndinia*, and *Terana*.

##### Notes.

The family Phanerochaetaceae was established by Jülich with the genus *Phanerochaete* as the type genus. This family belongs to the phlebioid clade within the order Polyporales and causes white rot (Larsson 2007; Binder et al. 2013; Miettinen et al. 2016; Justo et al. 2017). In the current study, twenty-seven genera are accepted in Phanerochaetaceae, including two new genera of the present study of *Paradonkia* and *Neodonkiella*.

#### ﻿ Paradonkia

Taxon classificationFungiPolyporalesPhanerochaetaceae

Y. Xu & C.L. Zhao gen. nov.

B7EC1A02-5AF5-5D23-92DF-9504B2DF7895

856347

##### Type species.

*Paradonkiafarinacea* Y. Xu & C.L. Zhao.

##### Etymology.

*paradonkia* (Lat.): “*para*” and “*donkia*” refer to a close phylogenetic relationship with the genus *Donkia*.

##### Description.

Basidiomata annual, resupinate, adnate. Hymenial surface farinaceous, pale cream to gray cream. Hyphal system monomitic; generative hyphae with both simple septa (more frequent) and clamp connections, colorless. Subicular hyphae colorless, thick-walled. Crystals abundant, crowded at hymenial layer and subiculum. Cystidia and cystidioles absent. Basidia clavate, thin-walled, 4-sterigmate. Basidiospores ellipsoid, colorless, thin-walled, smooth, IKI–, CB–.

##### Notes.

In our phylogenetic analysis (Fig. 1), *Paradonkia* is identified as a monophyletic group typified by *P.farinacea.* The new genus *Paradonkia* falls within the family Phanerochaetaceae (Polyporales) and is closely related to *Donkia.* The genus *Donkia* is distinguished from *Paradonkia* by its pileate basidiomata with a white to cream context, and cinnamon to orange-brown, odontoid to hydnoid hymenophore (Nakasone 1990; Chen et al. 2021).

#### ﻿ Paradonkia farinacea

Taxon classificationFungiPolyporalesPhanerochaetaceae

Y. Xu & C.L. Zhao sp. nov.

4EF048C4-BA8D-5AE8-81F1-7D7B083F0615

856348

Figs 3 , 4

##### Diagnosis.

Differs from other species by the farinaceous basidiomata with the pale cream to gray cream hymenial surface, a monomitic hyphal system, cystidia and cystidioles absent, narrowly clavate basidia, ellipsoid basidiospores.

##### Holotype.

China • Yunnan Province, Qujing, Zhanyi District, Lingjiao Town, Xiajia Village, 25°58'N, 103°47'E, altitude 2000 m, on the fallen angiosperm branch, leg. C.L. Zhao, 6 March 2023, CLZhao 27184 (SWFC).

##### Etymology.

*farinacea* (Lat.): refers to the holotype having a farinaceous hymenial surface.

##### Fruiting body.

Basidiomata annual, resupinate, adnate, without odor or taste when fresh, farinaceous, upon drying, up to 6.5 cm long, 1.3 cm wide, 110–180 μm thick. Hymenial surface smooth, white to cream when fresh, pale cream to gray cream when dry, unchanged in KOH. Sterile margin narrow, gray cream, 1 mm wide.

**Figure 3.**
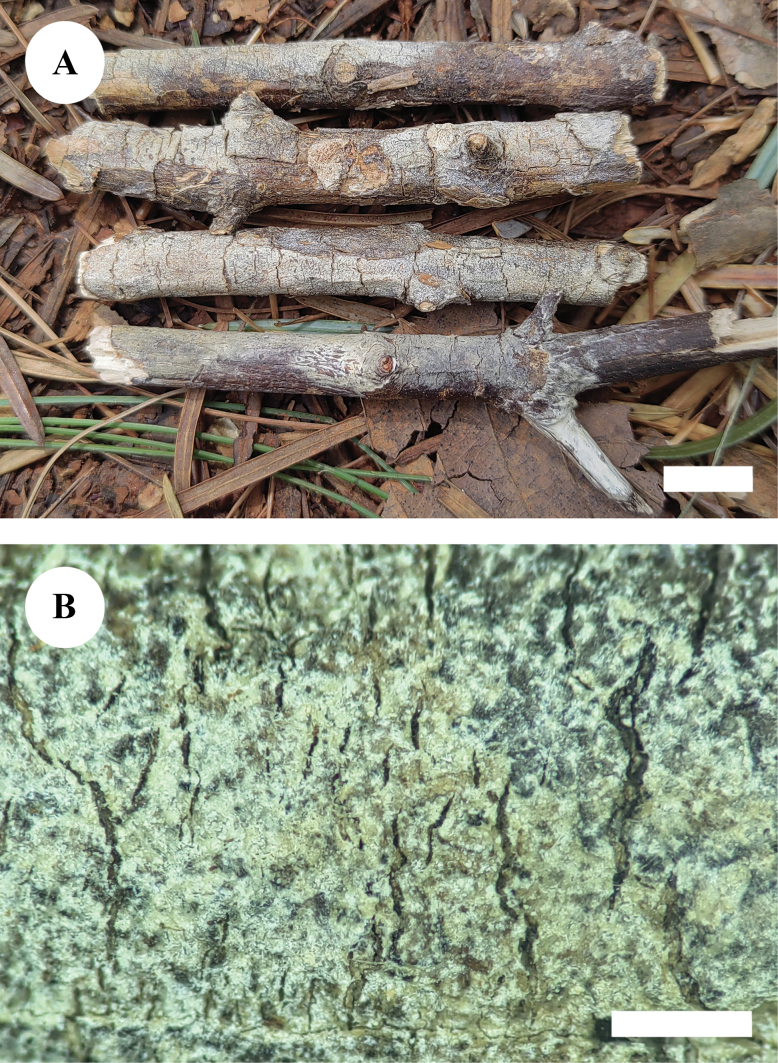
Basidiomata of *Paradonkiafarinacea* in general and detailed views (CLZhao 27184, holotype). Scale bars: 1 cm (**A**); 1 mm (**B**).

##### Hyphal system.

Monomitic, generative hyphae with simple septa and clamp connections, IKI–, CB–; tissues unchanged in KOH. Subicular hyphae mainly horizontal, colorless, thick-walled, slightly flexuous, rarely branched, interwoven, 6.0–7.5 μm in diameter. Crystals abundant, crowded. Subhymenium indistinct, hyphae in this layer similar to subicular hyphae.

**Figure 4.**
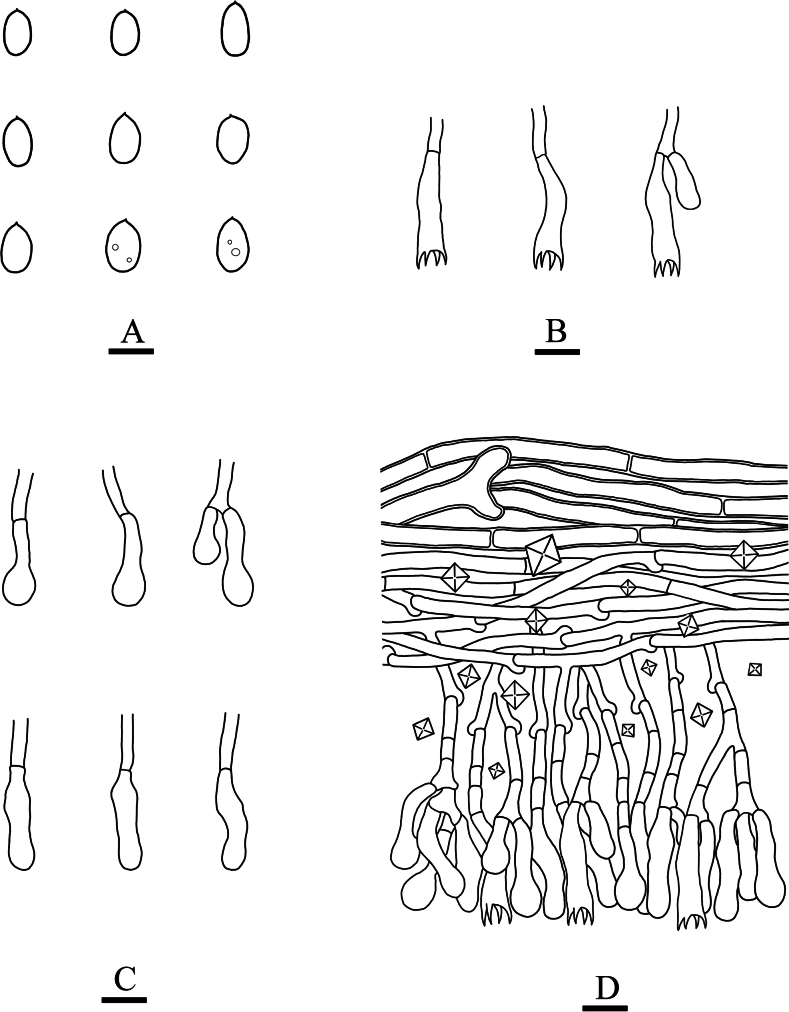
Microscopic structures of *Paradonkiafarinacea* (holotype, CLZhao 27184) **A** basidiospores **B** basidia **C** basidioles **D** a section of the fruit body. Scale bars: 5 µm (**A**); 10 µm (**B–D**); 10 × 100 Oil.

##### Hymenial layer.

Generative hyphae short-celled, colorless, 3–4 μm in diameter, thin- to slightly thick-walled. Crystals abundant, crowded. Cystidia and cystidioles absent. Basidia narrowly clavate, slightly flexuous, thin-walled, with four sterigmata and a simple septum, 25–29 × 4.5–6.5 μm. Basidioles similar to basidia in shape, but slightly smaller.

##### Basidiospores.

Ellipsoid, colorless, thin-walled, smooth, occasionally with oil drops, IKI–, CB–, 4–6(–6.5) × (2.5–)3–4(–4.5) μm, L = 4.87 μm, W = 3.37 μm, Q = 1.45 (n = 30/1).

##### Additional specimen examined

**(*paratype*).** • Yunnan Province, Qujing, Zhanyi District, Lingjiao Town, Xiajia Village, 25°58'N, 103°47'E, altitude 2000 m, on the fallen angiosperm branch, leg. C.L. Zhao, 6 March 2023, CLZhao 27221 (SWFC).

#### ﻿ Neodonkiella

Taxon classificationFungiPolyporalesPhanerochaetaceae

Y. Xu & C.L. Zhao gen. nov.

ACA6CA5C-2B70-5A92-9382-96F0C4007528

856349

##### Type species.

*Neodonkiellayinjiangensis* Y. Xu & C.L. Zhao.

##### Etymology.

*Neodonkiella* (Lat.): “*Neo*” and “*donkiella*” refer to the new genus’s molecular systematic similarity to the genus *Donkiella*.

##### Description.

Basidiomata annual, resupinate, adnate, soft coriaceous. Hymenial surface smooth, white to pale cream. Hyphal system monomitic; generative hyphae with both simple septa and clamp connections, colorless. Subicular hyphae colorless, thick-walled. Crystals abundant, crowded at hymenial layer and subiculum. Leptocystidia numerous in the hymenium. Cystidioles absent. Basidia clavate, thin-walled, 4-sterigmate. Basidiospores ellipsoid, colorless, thin-walled, smooth, IKI–, CB–.

##### Notes.

In our phylogenetic analysis (Fig. 1), the new genus *Neodonkiella* was identified as a monophyletic group typified by *P.yinjiangensis*. The new taxon *Neodonkiella* falls within the family Phanerochaetaceae (Polyporales) and is closely related to the genus *Donkiella. Donkiella* is distinguished from *Neodonkiella* by its generative hyphae with simple septa only (Dong et al. 2024).

#### ﻿ Neodonkiella yinjiangensis

Taxon classificationFungiPolyporalesPhanerochaetaceae

Y. Xu & C.L. Zhao sp. nov.

6B9B7CA3-328A-5861-87E3-BF2A4EF8D17C

856350

Figs 5 , 6

##### Diagnosis.

Differs from other species by pale white to pale cream hymenial surface, a monomitic hyphal system, slightly flexuous leptocystidia, narrowly clavate basidia, and ellipsoid basidiospores.

##### Holotype.

China • Yunnan Province, Dehong, Yingjiang County, Tongbiguan Provincial Nature Reserve, 23°48'N, 97°38'E, altitude 1500 m, on the fallen angiosperm branch, leg. C.L. Zhao, 19 July 2023, CLZhao 30585 (SWFC).

##### Etymology.

*yingjiangensis* (Lat.): refers to the locality (Yingjiang County) of the type specimen.

##### Fruiting body.

Basidiomata annual, resupinate, slightly adnate, without odor or taste when fresh, soft coriaceous upon drying, up to 3.5 cm long, 0.7 cm wide, 50–100 μm thick. Hymenial surface smooth, white when fresh, white to pale cream when dry, unchanged in KOH. Sterile margin narrow, white, up to 0.5 mm wide.

##### Hyphal system.

Monomitic; generative hyphae with simple septa and clamp connections, IKI–, CB–; tissues unchanged in KOH. Subicular hyphae colorless, thick-walled, straight, slightly branched, interwoven, 3–4 μm in diameter. Crystals abundant, crowded. Subhymenium indistinct, hyphae in this layer similar to subicular hyphae.

**Figure 5.**
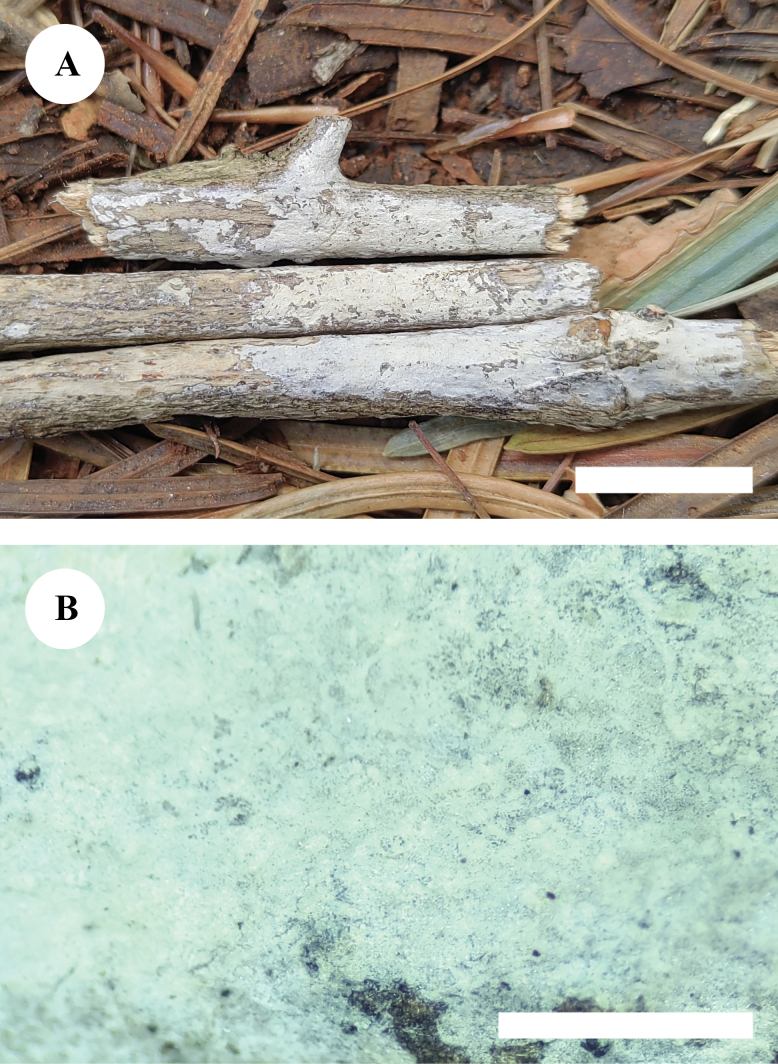
Basidiomata of *Neodonkiellayinjiangensis* in general and detailed views (CLZhao 30585, holotype). Scale bars: 1 cm (**A**); 1 mm (**B**).

##### Hymenial layer.

Generative hyphae vertical, short-celled, colorless, thin-walled, 2–3 μm in diameter. Crystal abundant, crowded. Leptocystidia colorless, thin-walled, slightly flexuous, smooth, sometimes with small oil drops, numerous in the hymenium, 25–32 × 2.5–4 μm. Basidia narrowly clavate, slightly flexuous, thin-walled, with four sterigmata and a simple septum, 18–23 × 4–5 μm. Basidioles similar to basidia in shape, but slightly smaller.

**Figure 6.**
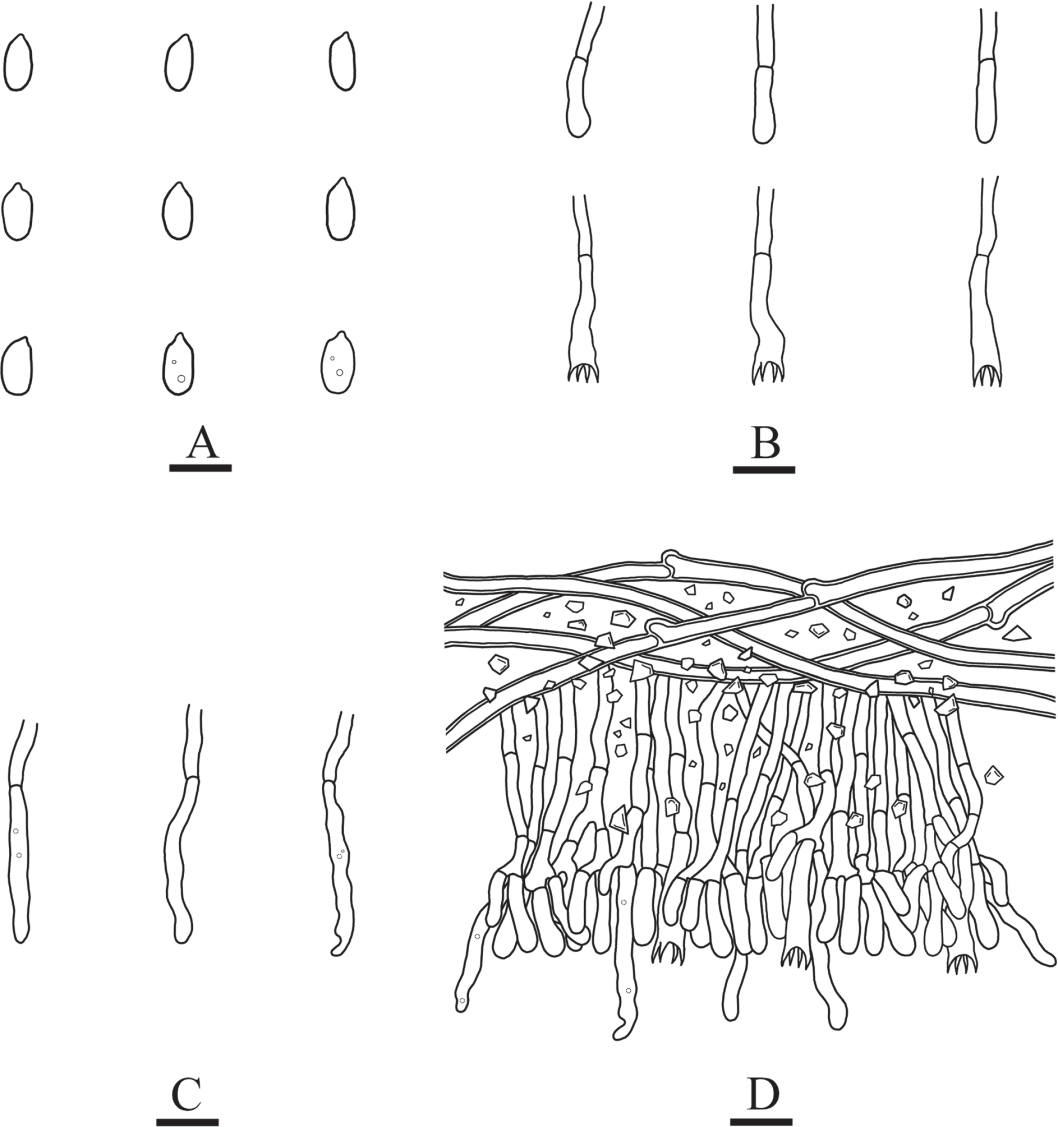
Microscopic structures of *Neodonkiellayinjiangensis* (holotype, CLZhao 30585) **A** basidiospores **B** basidia & basidioles **C** leptocystidia **D** a section of the fruit body. Scale bars: 5 µm (**A**); 10 µm (**B–D**); 10 × 100 Oil.

##### Basidiospores.

Ellipsoid, colorless, thin-walled, smooth, occasionally with small oil drops, IKI–, CB–, (3–)3.5–5 × (1.5–)2–2.5 μm, L = 4.1 μm, W = 2.2 μm, Q = 1.89 (n = 30/1).

#### ﻿ Phanerochaete

Taxon classificationFungiPolyporalesPhanerochaetaceae

P. Karst.

33C66C88-D2DF-5A2B-8B4B-91E88E6C8B37

##### Type species.

*Phanerochaetealnea* (Fr.) P. Karst.

##### Notes.

In our phylogenetic analysis (Fig. 2), *Phanerochaete* was recovered as a monophyletic with strong support of 59 species, including the three new species (*Phanerochaetealbocremea*, *P.fissurata*, and *P.punctata*) presented here. The basidiomata of *Phanerochaete* s.s. are typically membranaceous, in which the hymenophore is usually smooth, but tuberculate, grandinioid, odontioid to hydnoid or even poroid hymenophore occur in some species. Microscopically, *Phanerochaete* is characterized by having mostly a monomitic hyphal system with ordinarily simple septa hyphae and clavate basidia. Cystidia present in many species, which may be naked or encrusted, and often with thin walls. The colorless subiculum is present in most species, but a brownish subiculum also occurs (Chen et al. 2021; Deng et al. 2024).

#### ﻿ Phanerochaete albocremea

Taxon classificationFungiPolyporalesPhanerochaetaceae

Y. Xu & C.L. Zhao sp. nov.

A185A80F-712D-56A7-BEBB-912396513A10

856147

Figs 7 , 8

##### Diagnosis.

Differs from other species in the soft coriaceous basidiomata and white to pale cream hymenial surface, a monomitic hyphal system, clavate basidia, and narrowly ellipsoid basidiospores.

##### Holotype.

China • Yunnan Province, Zhaotong, Wumengshan National Nature Reserve, 28°03'N, 104°20'E, altitude 1500 m, on the fallen angiosperm branch, leg. C.L. Zhao, 28 August 2023, CLZhao 32235 (SWFC).

##### Etymology.

*albocremea* (Lat.): refers to the holotype having a white to pale cream hymenial surface.

##### Fruiting body.

Basidiomata annual, resupinate, adnate, without odor or taste when fresh, soft coriaceous upon drying, up to 5.2 cm long, 1.1 cm wide, 100–150 μm thick. Hymenial surface smooth, white when fresh, white to pale cream when dry, unchanged in KOH. Sterile margin white, fibrous, up to 2 mm wide.

**Figure 7.**
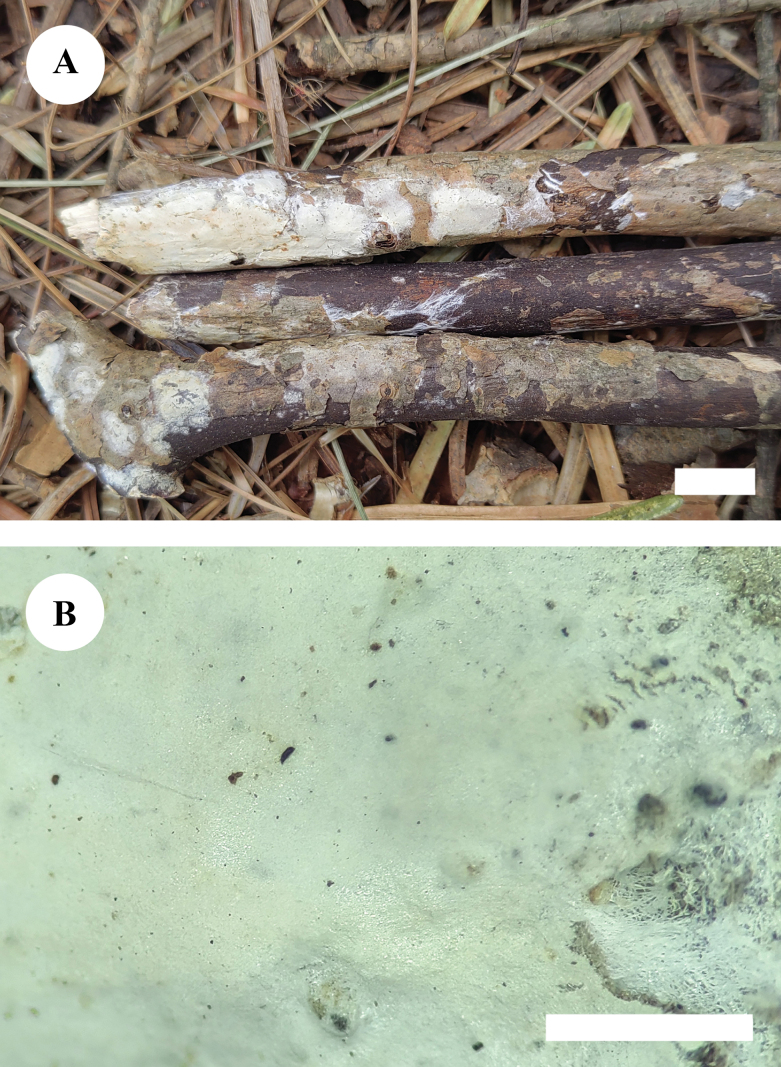
Basidiomata of *Phanerochaetealbocremea* in general and detailed views (CLZhao 32235, holotype). Scale bars: 1 cm (**A**); 1 mm (**B**).

##### Hyphal system.

Monomitic; generative hyphae simple septa, IKI–, CB–; tissues unchanged in KOH. Subicular hyphae colorless, thin- to thick-walled, straight, interwoven, usually encrusted with crystals, 6.5–9.5 μm in diameter. Crystals abundant, crowded. Subhymenium indistinct, hyphae in this layer similar to subicular hyphae.

**Figure 8.**
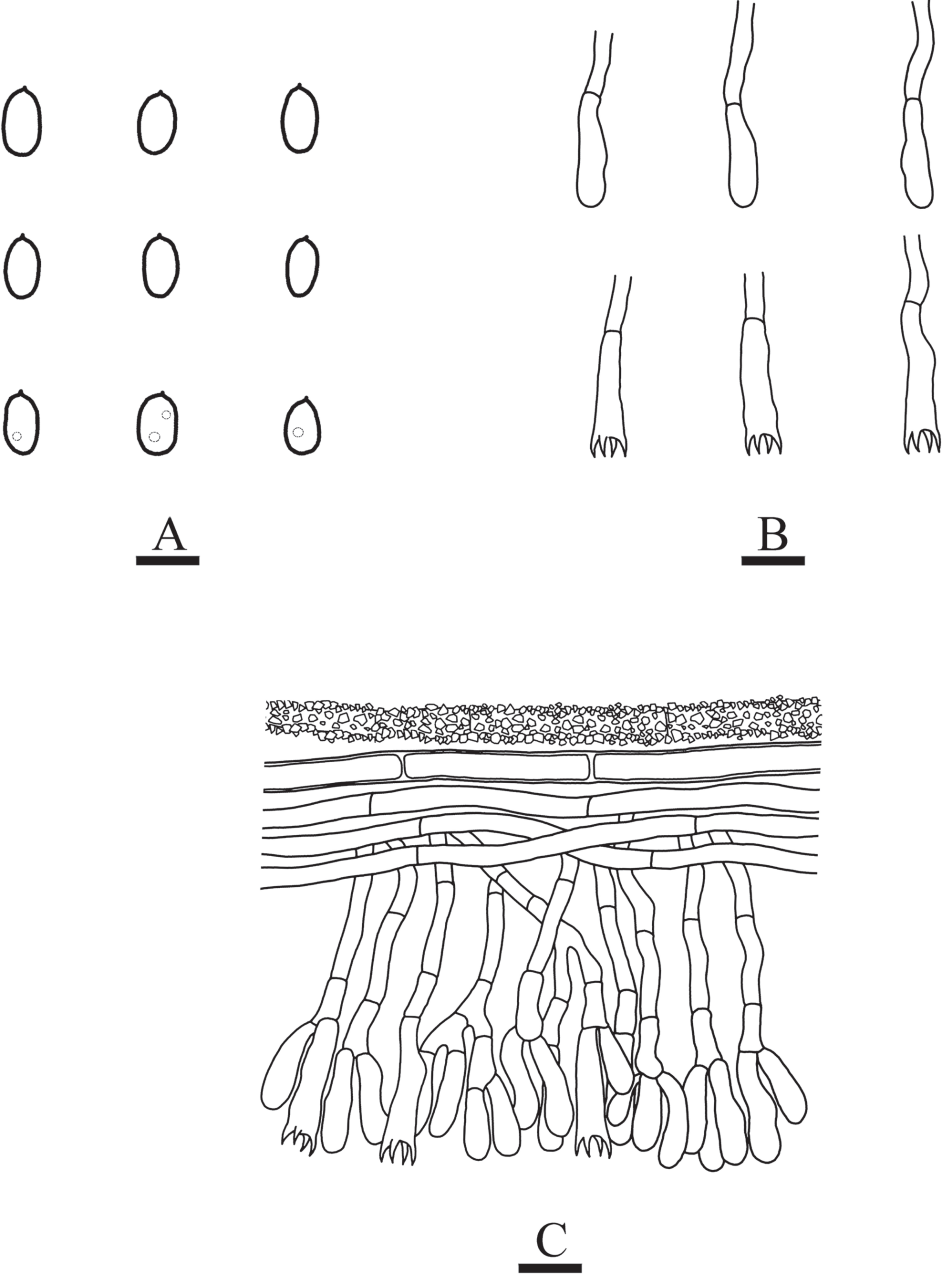
Microscopic structures of *Phanerochaetealbocremea* (holotype, CLZhao 32235) **A** basidiospores **B** basidia & basidioles **C** a section of the fruit body. Scale bars: 5 µm (**A**); 10 µm (**B–C**); 10 × 100 Oil.

##### Hymenial layer.

Generative hyphae vertical, short-celled, colorless, 3–5 μm in diameter, thin- to slightly thick-walled. Crystals abundant, crowded. Cystidia and cystidioles absent. Basidia clavate, slightly flexuous, thin-walled, with four sterigmata and a simple septum, 16–21 × 4–5.5 μm. Basidioles similar to basidia in shape, but slightly smaller.

##### Basidiospores.

Narrowly ellipsoid, colorless, thin-walled, smooth, occasionally with small oil drops, IKI–, CB–, 3.5–5 × 2–3(–3.5) μm, L = 4.30 μm, W = 2.69 μm, Q = 1.59 (n = 120/4).

##### Additional specimens examined

**(*paratypes*).** • Yunnan Province, Zhaotong, Wumengshan National Nature Reserve, 28°03'N, 104°20'E, altitude 1500 m, on the dead bamboo, leg. C.L. Zhao, 27 August 2023, CLZhao 31998; on the angiosperm stump, leg. C.L. Zhao, 27 August 2023, CLZhao 32032, CLZhao 32035 (SWFC).

#### ﻿ Phanerochaete fissurata

Taxon classificationFungiPolyporalesPhanerochaetaceae

Y. Xu & C.L. Zhao sp. nov.

25C02FC6-7975-56E0-992D-F54FCDF7E69C

856149

Figs 9 , 10

##### Diagnosis.

Differs from other species by the gray-brown and cracked hymenial surface, a monomitic hyphal system with brownish subicular hyphae, narrowly clavate basidia, and ellipsoid basidiospores.

##### Holotype.

China • Yunnan Province, Zhaotong, Daguan County, Wumengshan National Nature Reserve, 28°08'N, 103°58'E, altitude 1800 m, on the fallen angiosperm branch, leg. C.L. Zhao, 17 October 2023, CLZhao 35311 (SWFC).

##### Etymology.

*fissurata* (Lat.) refers to the holotype having a cracked hymenial surface.

##### Fruiting body.

Basidiomata annual, resupinate, slightly adnate, without odor or taste when fresh, soft coriaceous when fresh, hard coriaceous upon drying, up to 7.7 cm long, 1.8 cm wide, 50–120 μm thick. Hymenial surface smooth, pale cream when fresh, gray-brown when dry, unchanged in KOH, cracked. Sterile margin narrow, gray brown, up to 1.5 mm wide.

**Figure 9.**
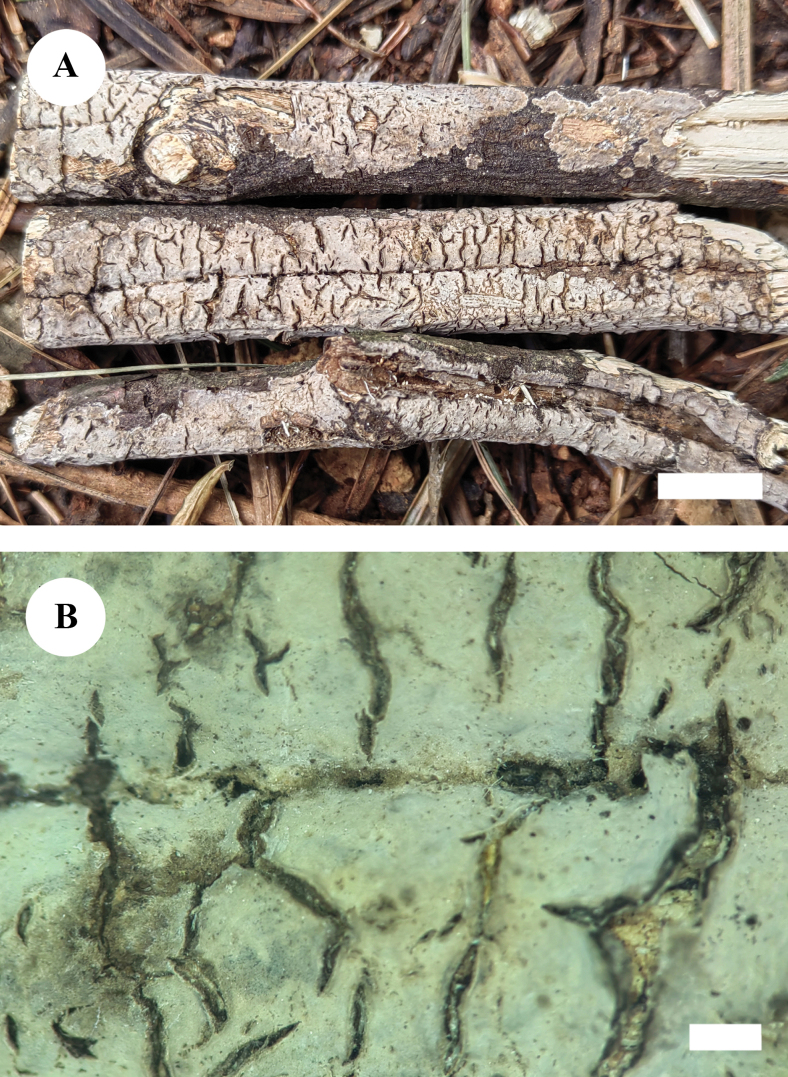
Basidiomata of *Phanerochaetefissurata* in general and detailed views (CLZhao 35311, holotype). Scale bars: 1 cm (**A**); 1 mm (**B**).

##### Hyphal system.

Monomitic; generative hyphae with simple septa, IKI–, CB–; tissues unchanged in KOH. Subicular hyphae brownish, thick-walled, slightly branched, interwoven, slightly flexuous, 3.5–5.5 μm in diameter. Subhymenium indistinct, hyphae in this layer similar to subicular hyphae.

**Figure 10.**
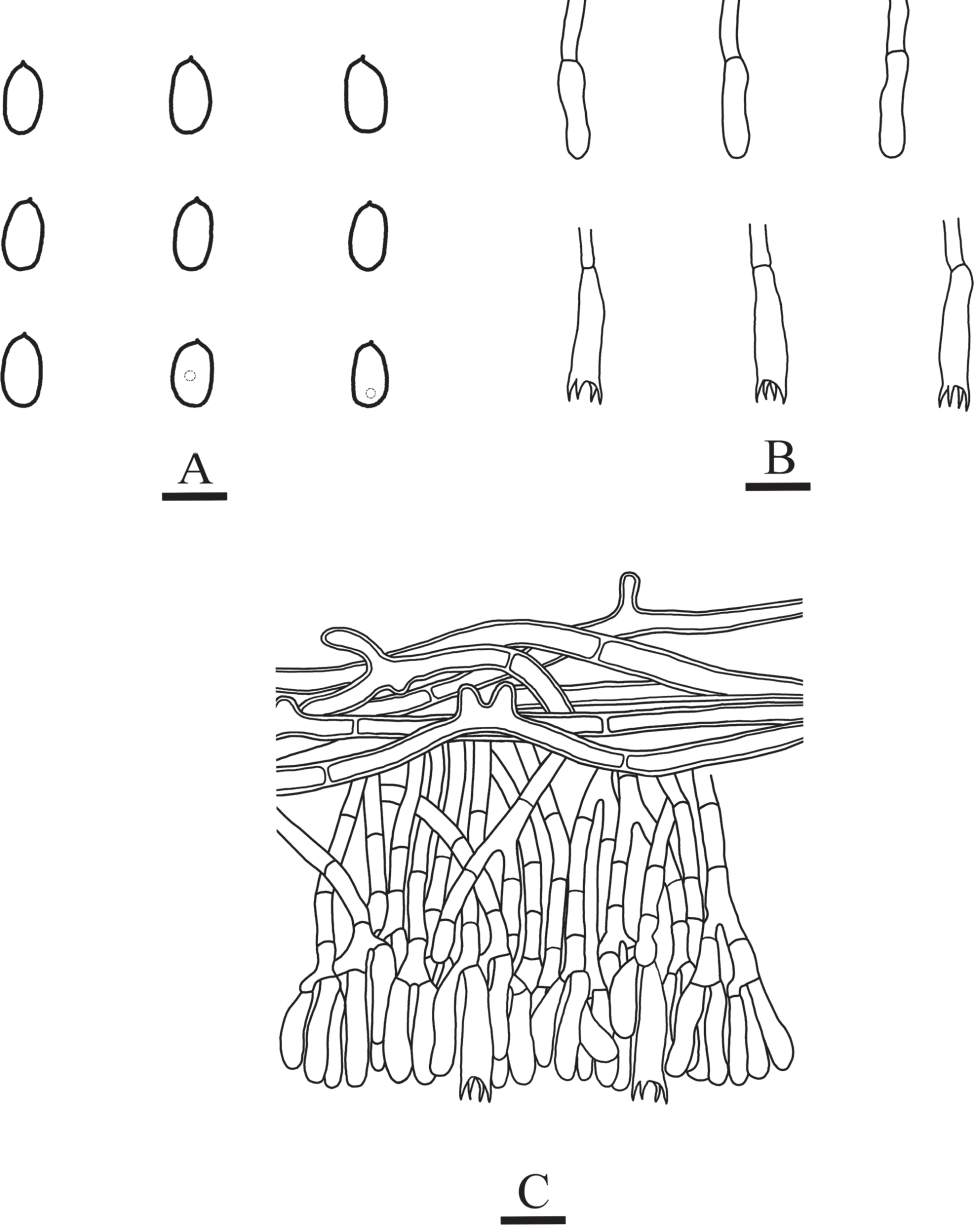
Microscopic structures of *Phanerochaetefissurata* (holotype, CLZhao 35311) **A** basidiospores **B** basidia & basidioles **C** a section of the fruit body. Scale bars: 5 µm (**A**); 10 µm (**B–C**); 10 × 100 Oil.

##### Hymenial layer.

Generative hyphae vertical, short-celled, colorless, 3–4.5 μm in diameter, thin- to slightly thick-walled. Cystidia and cystidioles absent. Basidia narrowly clavate, thin-walled, with four sterigmata and a simple septum, 17.5–21.5 × 3.5–5.5 μm. Basidioles in shape are similar to basidia, but slightly smaller.

##### Basidiospores.

Ellipsoid, colorless, thin-walled, smooth, occasionally with small oil drops, IKI–, CB–, 4–5.5(–6) × 2–3(–3.5) μm, L = 4.70 μm, W = 2.43 μm, Q = 1.85–2.02 (n = 60/2).

##### Additional specimen examined

**(*paratype*).** • Yunnan Province, Zhaotong, Daguan County, Wumengshan National Nature Reserve, 28°08'N, 103°58'E, altitude 1800 m, on the fallen angiosperm branch, leg. C.L. Zhao, 17 October 2023, CLZhao 35321 (SWFC).

#### ﻿ Phanerochaete punctata

Taxon classificationFungiPolyporalesPhanerochaetaceae

Y. Xu & C.L. Zhao sp. nov.

406033B2-4231-552F-AA80-91D5BC8E3298

856148

Figs 11 , 12

##### Diagnosis.

Differs from other species in thin basidiomata and white to pale buff hymenial surface, a monomitic hyphal system, cylindrical to subfusiform leptocystidia, clavate basidia, and ellipsoid basidiospores.

##### Holotype.

China • Yunnan Province, Dehong, Yingjiang County, Tongbiguan Provincial Nature Reserve, 23°48'N, 97°38'E, altitude 1500 m, on the fallen angiosperm branch, leg. C.L. Zhao, 19 July 2023, CLZhao 30512 (SWFC).

##### Etymology.

*punctata* (Lat.) refers to the holotype having punctate basidiomata.

##### Fruiting body.

Basidiomata annual, resupinate, adnate, without odor or taste when fresh, membranaceous upon drying, up to 6.3 cm long, 1.7 cm wide, 40–100 μm thick. Hymenial surface thin, white when fresh, white to pale buff when dry, unchanged in KOH. Sterile margin narrow, fibrous, white, up to 0.5 mm wide.

**Figure 11.**
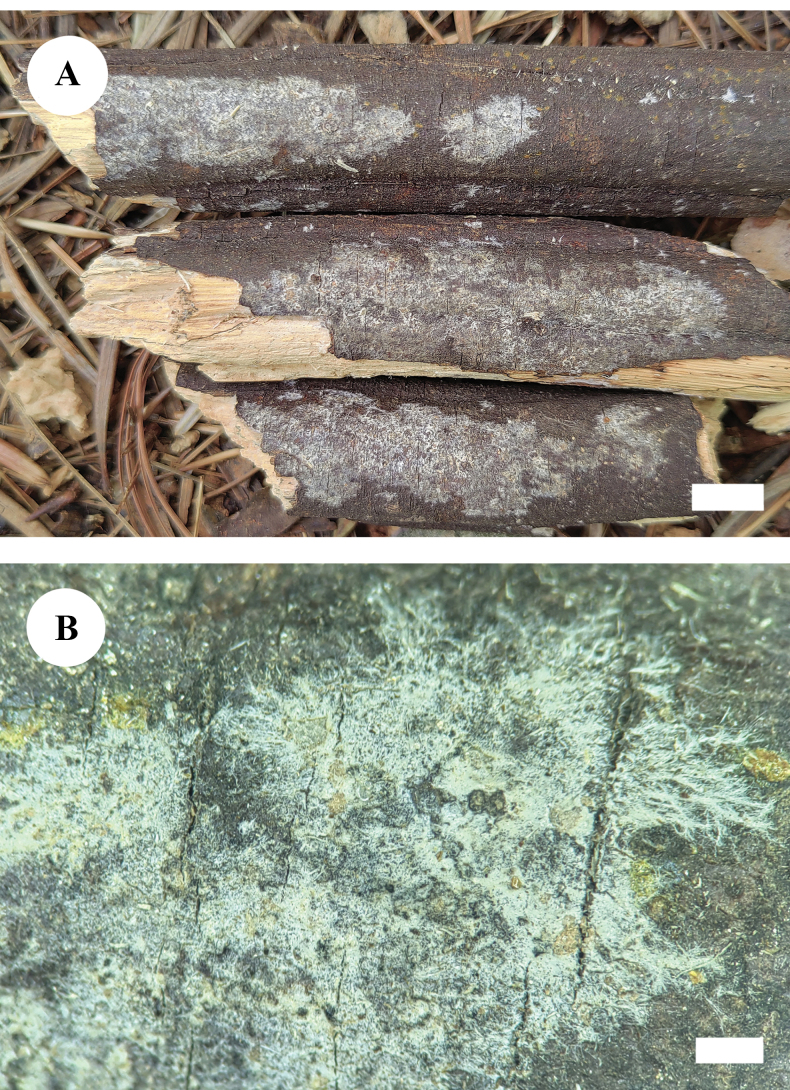
Basidiomata of *Phanerochaetepunctata* in general and detailed views (CLZhao 30512, holotype). Scale bars: 1 cm (**A**); 1 mm (**B**).

##### Hyphal system.

Monomitic; generative hyphae mostly simple septate, rarely with single or double clamp connections, IKI–, CB–; tissues unchanged in KOH. Subicular hyphae colorless, thick-walled, straight, interwoven, 5.5–8.5 μm in diameter, presence of double clamp connections. Crystals abundant, crowded. Subhymenium indistinct, hyphae in this layer similar to subicular hyphae.

**Figure 12.**
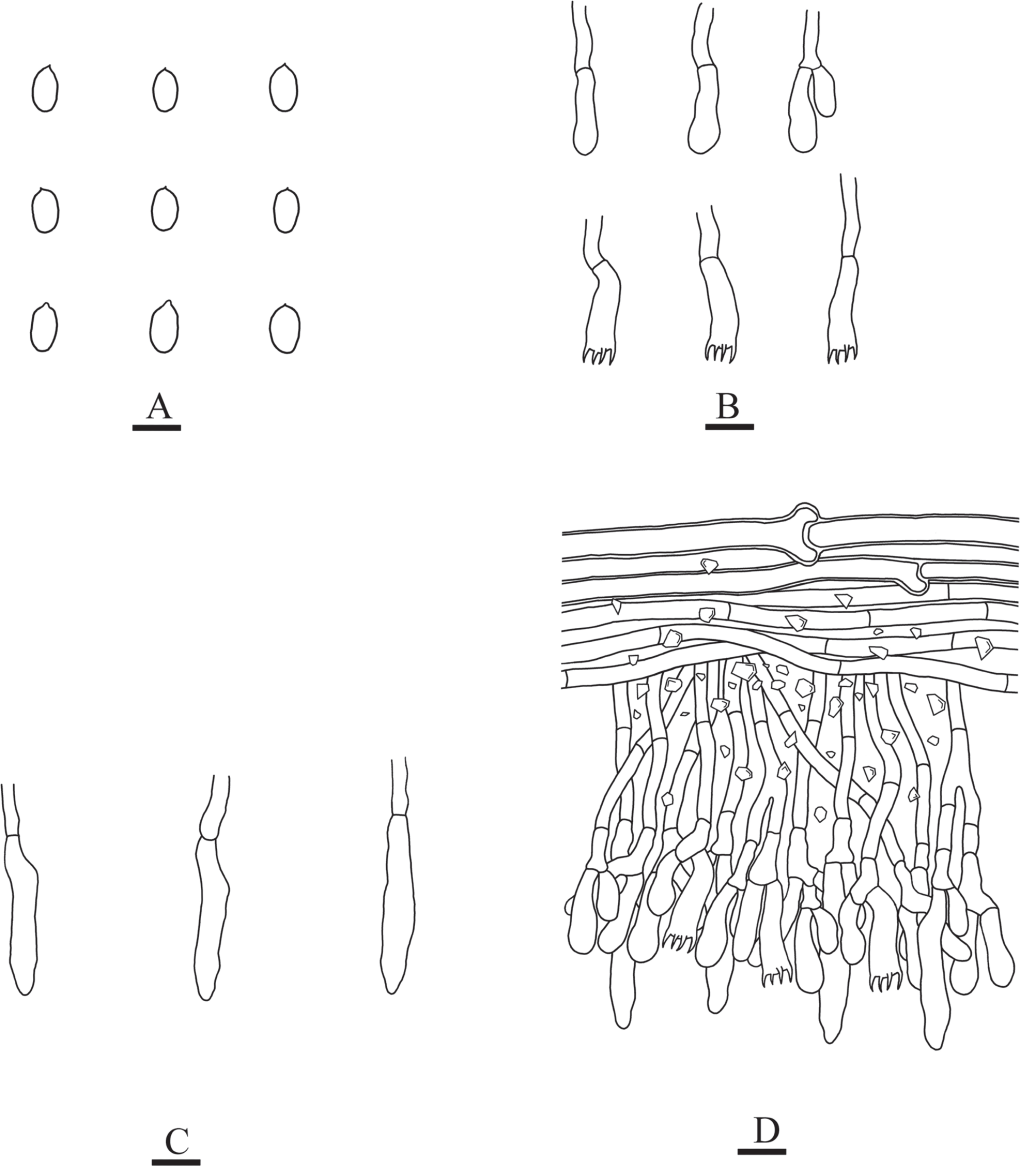
Microscopic structures of *Phanerochaetepunctata* (holotype, CLZhao 30512) **A** basidiospores **B** basidia & basidioles **C** leptocystidia **D** a section of the fruit body. Scale bars: 5 µm (**A**); 10 µm (**B–D**); 10 × 100 Oil.

##### Hymenial layer.

Generative hyphae vertical, short-celled, colorless, 3–4.5 μm in diameter, thin- to slightly thick-walled. Crystals abundant, crowded. Leptocystidia cylindrical to subfusiform, colorless, thin-walled, smooth, sometimes slightly flexuous, numerous, 30–37.5 × 4.5–7 μm. Basidia clavate, slightly flexuous, thin-walled, with four sterigmata and a simple septum, 18–22 × 5–7 μm. Basidioles similar to basidia in shape, but slightly smaller.

##### Basidiospores.

Ellipsoid, colorless, thin-walled, smooth; IKI–, CB–; 3.5–5(–5.5) × 2–3.5 μm, L = 4.29 μm, W = 2.79 μm, Q = 1.53 (n = 30/1).

##### Additional specimen examined

**(*paratype*).** China • Yunnan Province, Dehong, Yingjiang County, Tongbiguan Provincial Nature Reserve, 23°48'N, 97°38'E, altitude 1500 m, on the fallen angiosperm branch, leg. C.L. Zhao, 19 July 2023, CLZhao 30365 (SWFC).

## ﻿Discussion

In the present study, two new genera, *Paradonkia* and *Neodonkiella*, and five new species, *Paradonkiafarinacea*, *Neodonkiellayinjiangensis*, *Phanerochaetealbocremea*, *Phanerochaetefissurata* and *Phanerochaetepunctata* are described based on phylogenetic analyses and morphological characteristics.

*Phanerochaete* is widely distributed in the world and has extremely important research value. It was the 13^th^ most-cited fungus in 2011–2021, and it is the highest-cited fungus in basidiomycetes (Bhunjun et al. 2024). Phylogenetically, based on the combined ITS+nLSU sequence data (Figs 1, 2), it demonstrated that two new genera and the five new species were all nested in the family Phanerochaetaceae, in which *P.albocremea*, *P.fissurata* and *P.punctata* were nested in the genera *Phanerochaete* within the family Phanerochaetaceae of the order Polyporales (Basidiomycota).

Based on ITS+nLSU topology tree (Fig. 1), *Paradonkiafarinacea* was retrieved as a sister to *Donkiapulcherrima* (Berk. & M.A. Curtis) Pilát, and the species *Neodonkiellayinjiangensis* was sister to *Donkiellayunnanensis*. However, *Donkiapulcherrima* differs from *Paradonkiafarinacea* by its pileate basidiomata with white to cream context, cream to white with orange tones hymenial surface, and the presence of the multiple clamp connections on the context hyphae (Chen et al. 2021). *Donkiellayunnanensis* J.H. Dong & C.L. Zhao is distinguished from *Neodonkiellayinjiangensis* by its membranous basidiomata, generative hyphae with simple septa, and wider basidiospores (4.2–6 × 2.5–3.2 µm vs. 3.5–5 × 2–2.5 µm; Dong et al. 2024).

Based on ITS+nLSU topology tree (Fig. 2), *Phanerochaetealbocremea* formed a monophyletic lineage and was closely related to *P.porostereoides* and *P.fusca*. *P.fissurata* was retrieved as a sister to *P.cinerea*, and *P.punctata* was sister to *P.hainanensis.* However, *P.porostereoides* differs from *P.albocremea* by its brown to dark brown hymenial surface, brown subicular hyphae, and longer basidia (23–35 × 4–5.3 µm vs. 16–21 × 4–5.5 μm; Liu and He 2016). *Phanerochaetefusca* differs from *P.albocremea* by its dark brown hymenial surface, brown subicular hyphae, longer basidia (22–50 × 5–6 µm vs. 16–21 × 4–5.5 μm) and bigger basidiospores (5.7–7.3 × 3–3.5 μm vs. 3.5–5 × 2–3 μm; Wu et al. 2018). *Phanerochaetecinerea* differs from *P.fissurata* by its gray to grayish brown hymenial surface and with many small patches (Xu et al. 2020). *Phanerochaetehainanensis* is distinguished from *P.punctata* by its orange hymenophore, all generative hyphae without clamp connections, longer subulate to subcylindrical cystidia (35–70 × 3–7 μm vs. 30–37.5 × 4.5–7 μm; Boonmee et al. 2021).

Morphologically, *Phanerochaetealbocremea* resembles *P.rhizomorpha* by having a cream hymenial surface. However, *P.rhizomorpha* differs from *P.albocremea* by its membranous basidiomata, and longer basidia (25–28 × 4–5 μm vs. 16–21 × 4–5.5 μm; Chen et al. 2021). *Phanerochaetefissurata* is similar to *P.thailandica* by having brown subicular hyphae, but the latter having both bigger basidia (25–38 × 5–7 μm vs. 17.5–21.5 × 3.5–5.5 μm) and basidiospores (7–8 × 4–4.5 µm vs. 4–5.5 × 2–3 μm; Sádlíková and Kout 2017). *Phanerochaetepunctata* resembles *P.sinensis* by having clavate basidia. However, *P.sinensis* is distinguished from *P.punctata* by its white to orange hymenophore and longer leptocystidia (35–50 × 4–6 µm vs. 30–37.5 × 4.5–7 μm; Xu et al. 2020).

Corticioid fungi are a large group of wood-inhabiting fungi with simpler basidiomata and fewer distinguishing morphological features when compared with polypores and mushrooms, but its species and phylogenetic diversity are even higher than polypores but less intensively studied (Larsson et al. 2004; Binder et al. 2005; Bernicchia and Gorjón 2010; Dai 2011; Sun et al. 2020). A large amount of corticioid taxa have not been discovered and descSunribed worldwide, especially in the subtropical and tropical areas (Yang et al. 2023; Zhou et al. 2024). As shown in this study and earlier ones (Volobuev et al. 2015; Chen et al. 2018; Ordynets et al. 2018; Wu et al. 2022b; Wang et al. 2023), DNA sequence data are very useful in exploring cryptic taxa and diversity of corticioid fungi. Thus, in order to understand the diversity, phylogeny, and evolution of fungi, future taxonomic and phylogenetic work should focus more on the corticioid group by using both molecular and morphological characters (Xu et al. 2020).

## Supplementary Material

XML Treatment for Phanerochaetaceae

XML Treatment for Paradonkia

XML Treatment for Paradonkia farinacea

XML Treatment for Neodonkiella

XML Treatment for Neodonkiella yinjiangensis

XML Treatment for Phanerochaete

XML Treatment for Phanerochaete albocremea

XML Treatment for Phanerochaete fissurata

XML Treatment for Phanerochaete punctata

## ﻿Additional information

### Author ORCIDs

Ying Xu https://orcid.org/0009-0004-1599-0630

Yang Yang https://orcid.org/0000-0001-8054-948X

Xin Yang https://orcid.org/0009-0009-7755-0581

Daxiang Chen https://orcid.org/0009-0001-2309-1991

Wen Zheng https://orcid.org/0009-0005-9996-4284

Kaize Shen https://orcid.org/0000-0002-0441-4257

Sicheng Zhang https://orcid.org/0009-0003-6287-9823

### Data availability

All of the data that support the findings of this study are available in the main text.
